# If not When, then Where? Ignoring Temporal Information Eliminates Reflexive but not Volitional Spatial Orienting

**DOI:** 10.3390/vision1020012

**Published:** 2017-05-06

**Authors:** Kaitlin E. W. Laidlaw, Alan Kingstone

**Affiliations:** 1 Brain and Mind Institute, Western University, London, ON N6A 5B7, Canada; 2 Psychology Department, University of British Columbia, Vancouver, BC V6T 1Z4, Canada

**Keywords:** spatial attention, temporal attention, attentional orienting, reflexive attention orienting, volitional attention orienting, symbolic cue, visual attention

## Abstract

A tremendous amount of research has been devoted to understanding how attention can be committed to space or time. Until recently, relatively little research has examined how attention to these two domains combine. The present study addressed this issue. We examined how implicitly manipulating whether participants used a cue to orient attention in time impacts reflexive or volitional shifts in spatial attention. Specifically, participants made speeded manual responses to the detection of a peripherally presented target that appeared either 100, 500, or 1000 ms after the onset of a central cue. Cues were either spatially non-predictive arrows (*p = 0.50*) or spatially-predictive (*p* = 0.80) letter cues. Whereas arrow cues can reflexively orient spatial attention even when non-predictive of a target’s spatial location, letters only orient spatial attention when they reliably predict a target location, i.e., the shift is volitional. Further, in one task, a target was presented on every trial, thereby encouraging participants to use the temporal information conveyed by the cue to prepare for the appearance of the target. In another task, 25% of trials contained no target, implicitly discouraging participants from using the cue to direct attention in time. Results indicate that when temporal information is reliable and therefore volitionally processed, then spatial cuing effects emerge regardless of whether attention is oriented reflexively or volitionally. However, when temporal information is unreliable, spatial cuing effects only emerge when spatial cue information is reliable, i.e., when spatial attention is volitionally shifted. Reflexive cues do not elicit spatial orienting when their temporal utility is reduced. These results converge on the notion that reflexive shifts of spatial attention are sensitive to implicit changes in a non-spatial domain, whereas explicit volitional shifts in spatial attention are not.

## 1. Introduction

Human visual attention can be oriented in both space and time [1,2]. In so doing, people can improve processing of their visual world not only to particular locations, but also during expected time periods [3,4,5,6,7,8]. In the Posner cuing paradigm [9,10], participants make speeded manual responses to a peripherally presented target. As Posner demonstrated, if the onset of the target is validly cued by a brief change in luminance at the target’s spatial location, then attention will be reflexively directed to the target’s location, quickly facilitating processing and speeding responses. If, however, the target is invalidly cued, or preceded by the same flash at an opposing spatial location, responses will initially be delayed because attention is misdirected by the cue and must be reallocated to the target’s location upon its appearance. The difference in response times (RT) between validly and invalidly cued trials is known as the spatial cuing effect. A number of visual properties, including motion transients, changes in luminance, and the presence of a unique object singleton, have all been proposed to reflexively shift attention to their location, even when they do not predict the location of an upcoming target [11,12,13].

### 1.1. Spatial Orienting to Central Cues can be Reflexive as well as Volitional

For some time, attentional orienting was considered to be either a reflexive or a volitional process (also referred to as exogenous or endogenous, automatic or controlled, bottom-up or top-down, respectively) [9,14]. The dichotomy, it seemed, was relatively straightforward: whereas peripheral visual changes could automatically re-orient attention to their location, centrally presented symbolic cues needed to orient attention via a controlled, volitional process.

With the turn of the century, however, researchers recognized that reflexive orienting can also be elicited by certain centrally presented symbolic cues, for example arrows or eye gaze [15,16,17,18,19,20,21], but see [22] for boundary conditions. Many have reported that reflexive orienting in response to central cues may be qualitatively different than to peripheral exogenous cues [15], such that peripheral cues will trigger orienting reflexively even in young infants [23] whereas reflexive orienting to centrally presented cues requires the development of an over-learned association between a cue and its ascribed directionality [24,25,26,27]. Nevertheless, the two types of cues share many fundamental similarities, with both triggering rapid orienting, even when the cue does not predict an upcoming target location [19,21,25,28,29].

Of course, attention can also be oriented volitionally in response to a central cue. To study this effect independently of any non-volitional orienting, an experimenter will use a central cue that does not already possess an association with a particular direction, so that when in the experiment the cue provides predictive information concerning the location of an upcoming target, only volitional attention is engaged. For instance, letters or colors are not strongly associated with any particular direction [30]; so if an observer is informed that during an experiment such a non-spatial feature reliably predicts that a target will appear at a particular location, then that observer can shift attention volitionally to the cued location in anticipation of the appearance of the cued target. This once again results in a spatial cuing effect: faster RTs for the cued (attended) target than to the uncued or invalidly cued target.

### 1.2. Attention in the Temporal Domain

This relation between a cue and a target need not be restricted to the spatial domain. Cuing effects are observed when attention is committed to an object’s non-spatial features, such as its color [31,32], or expected time of onset [33]. A real world example of both of these non-spatial instances occurs when a traffic light changes to amber, as it signals that a red light will shortly appear and people can shift their attention accordingly. Interestingly, it takes time to orient attention, especially when it is committed volitionally. This is readily apparent when one prepares for the time that a target will appear [33], with RT declining (getting faster) as the time between the cue and target interval increases from 0 to about a second. This decline in RT is known as a foreperiod effect [34]. Note that the foreperiod effect is understood to reflect an observer directing attention volitionally to the time when a target is expected to occur based on the information provided by a preceding cue, much as an amber light forewarns the onset of a red light [4,35,36,37].

Often cuing paradigms use multiple cue-target intervals (called stimulus onset asynchronies, or SOAs), each equally likely to occur. This creates an aging temporal distribution [38,39] which encourages the development of implicit expectancies concerning when a target will appear. An aging temporal distribution has the characteristic in which the probability of a target appearing increases as time passes after a cue’s onset. Consider an experiment in which a target occurs on every trial, and participants are presented with 100 trials at each of three cue-target SOAs. The probability of the target appearing following each SOA is determined by the total number of trials at that SOA divided by the total number of trials less any trials that have a shorter SOA. In this case, the target has a probability of 0.33 of appearing following the shortest SOA (100/300), a probability of 0.50 of appearing following the mid-length SOA (100/(300−100)), and a probability of 1 of appearing at the longest SOA (100/(300−200)). The predictability encourages participants to voluntarily entrain themselves to the cue’s temporal information, especially as more time passes after the cue’s onset when probabilities reach and exceed 0.50.

Foreperiod effects, a result of volitional temporal preparation, should not be confused with effects due to alertness, which instead are reflective of a general readiness to respond [40]. Typically, alertness is high when there is a strong relationship between the cue and the target (i.e., when the target frequently occurs following a cue), and low when many no-target trials are included (i.e., when the cue is not a reliable predictor that a target will appear) [41,42]. Low alertness will generally slow RT, whereas high alertness will have the opposite effect.

However, the inclusion of no-target catch trials, which decreases alertness, can also influence the probabilities of a target appearing following a given SOA. In this way, catch trials can also influence the foreperiod effect depending on how they are integrated into the paradigm [42]. Consider the same experiment above, but with an additional 100 catch trials in which no target appears, resulting in a total of 400 trials. Now, the probability of the target appearing at the shortest SOA is 0.25 (100/400), 0.33 at the mid-length SOA (100/(400−100)), and 0.5 at the longest SOA (100/(400−200)). The inclusion of catch trials should therefore not only result in a general increase in RT, but also a reduction in the foreperiod effect, because SOA is not a reliable predictor of the target’s appearance in time [41]. A reduced foreperiod effect following the addition of catch trials is consistent with a temporal reorienting hypothesis, which suggests that participants who may have oriented their attention to an early time period will reorient their attention to a later time point if no target appeared at the earlier SOA. Critically, however, attention will only be reoriented to a later time if the cue is a reliable predictor of the target appearing eventually. When catch trials are included, this instead implicitly leads participants to disengage from allocating their attention to time based on the unreliability by which the cue predicts if a target will occur [1,5,43,44,45,46,47].

### 1.3. The Influence of Attending to Time on Spatial Attention Effects

There is certainly no question that people can, and do, routinely combine temporal and spatial information, as when one makes an appointment for a particular time and place. However, whether the act of attending to the time of an expected event also engages spatial attention (and vice versa), is much less understood and has been the focus of recent investigation. Weinbach, Shofty, Gabay, and Henik [48] asked if volitional orienting in space and time can co-occur independently, and concluded that they could. In their study, a shape was presented centrally: the shape’s identity predicted when a target would most likely occur, while the color of the shape predicted where that target would likely occur. The results indicated both a temporal validity effect (RTs were faster when a target appeared at the validly versus invalidly cued time), and a spatial cuing effect (RTs were faster when a target appeared at the validly versus invalidly cued location). Critically, these effects did not interact, leading Weinbach and colleagues to suggest that volitionally allocating attention in time can occur independent of the volitional allocation of attention in space.

In contrast, that same year Hayward and Ristic [43] examined the effect of temporal and spatial attention, but on this occasion they were interested in the effect of temporal attention on reflexive spatial attention, e.g., when a central arrow cue points left or right, but does not predict where the target will appear. Recall that recent work has shown that when a cue is normally associated with a particular location (like an arrow) then spatial attention is shifted to the cued location even if, in the experiment, the cue does not reliably predict where the target will appear. Unlike Weinbach and colleagues’ [48] study, Hayward and Ristic [43] found that reflexive spatial orienting to a central arrow interacted with temporal attention, such that the spatial cuing effect grew in magnitude when participants were implicitly discouraged from attending to the time at which the target might occur. This was accomplished by the authors introducing a high number of catch trials into the study, thereby weakening the association that a cue reliably predicted the time of the target. However, in a follow-up study, Hayward and Ristic [46] introduced a more explicit manipulation of temporal attention, akin to the paradigm used by Weinbach and colleagues [48], and found that volitional temporal attention and reflexive spatial attention produced additive effects, i.e., were independently allocated.

In sum, recent work suggests that temporal attention, when it is explicitly cued, does not interact with volitional or reflexive spatial attention to a central cue. However, when temporal attention is implicitly manipulated by discouraging participants from expecting a target at a particular time (through the introduction of a number of catch trials), then temporal attention and spatial attention—that is, reflexive spatial attention – interact. What is unknown however, is whether this interaction between implicit temporal attention and reflexive spatial attention extends to volitional spatial attention. The present study addresses precisely this question.

### 1.4. The Present Study

In the present study, participants completed a cuing task with either an arrow cue or a letter cue. Whereas an arrow cue can reflexively orient spatial attention owing to an overlearned association between the arrow’s identity and a direction, a letter cue does not reliably direct spatial attention in a reflexive manner [30]. To encourage volitional orienting in response to the identity of the letter cue, the cue predicted the likely location of the target with an 80% probability. SOAs of 100, 500, and 1000 ms were used. For some participants, a target appeared during each trial, whereas for other participants, targets appeared on 75% of the trials (i.e., there were 25% catch trials). Although observers are capable of entraining to a cue’s temporal information in paradigms with or without catch trials, the inclusion of a high number of no-target trials serves to reduce the overall probability that any given cue will be followed by a target (see Table 1), thereby implicitly discouraging participants from volitionally orienting their attention in time [42,43,46]. On the other hand, in the absence of catch trials, the association between the cue and target’s appearance is perfectly reliable: if the target does not appear at the shortest SOA, then there is motivation to reorient attention to later SOAs. Thus, spatial attention could either be reflexively (arrow) or volitionally (letter) oriented, while the probability of the cue providing reliable temporal information concerning the target was manipulated implicitly by the inclusion or exclusion of catch trials.

Based on past work, we expect that manipulating temporal attention implicitly will interact with reflexive spatial attention (arrow cues), such that when attention is directed to the cue in order to extract temporal information concerning the upcoming appearance of the target, then spatial attentional orienting will also be affected [43]. Whether an implicit allocation of attention to a point in time (based on a cue’s information regarding the likely time of a target’s onset) has a similar effect on the volitional commitment of attention to space (letter cues) is unknown; and this is the key issue that the present study addresses. If the effects are similar, then we expect that an implicit change in temporal attention will interact with volitional spatial attention in a manner comparable to what has been observed with reflexive attention. On the other hand, to the extent that the explicit allocation of attention to space renders it separate from, and resistant to, implicit changes in temporal attention, volitional spatial orienting to letter cues should be independent of implicit changes in temporal attention. The end result will be that changes in temporal attention will have a very different effect on volitional spatial orienting compared to reflexive spatial orienting, such that volitional spatial orienting would be relatively unaffected by whether or not the letter cue provides reliable temporal information about the appearance of the target [43,46,48].

## 2. Methods

### 2.1. Participants

Seventy-two participants (48 females; 68 right handed; mean, M_age_ = 21.83 years; standard deviation, SD_age_ = 4.67 years) from the University of British Columbia participated for course credit or monetary remuneration. All had normal or corrected-to-normal vision. All gave informed written consent for inclusion prior to the start of the experiment. The study was conducted in accordance with the Declaration of Helsinki, and the protocol was approved by the University of British Columbia Behavioural Research Ethics Board (ID: H04-80767).

### 2.2. Stimuli and Procedure

**Figure 1:**
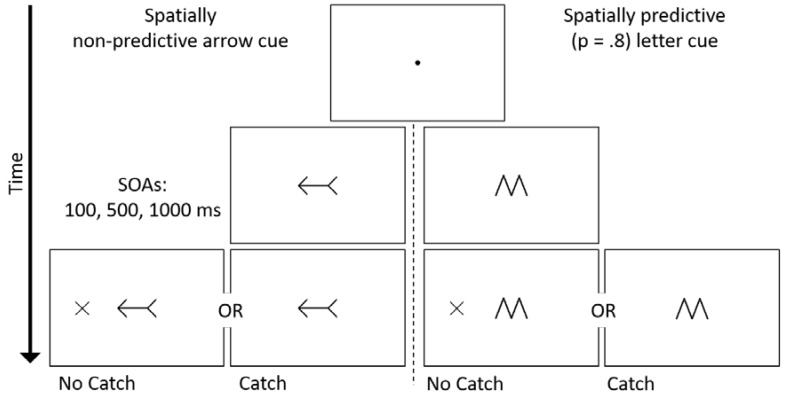
Schematic trial timeline, using either spatially non-predictive arrow cues or spatially predictive letter cues. The letter cue (M or W) correctly predicted the location of a target present trial 80% of the time. Half of the participants performed the task in the absence of catch trials. For the remainder of participants, 25% of trials did not contain a target and participants were required to withhold making a response. As an example, the timeline is presented for a spatially valid arrow cue; letter-direction association was counterbalanced across participants.

Stimuli were presented on a 16-inch cathode ray tube color monitor. Viewing distance was kept constant with a chin rest placed 50 cm from the screen. Stimuli were presented on a white background. The sequence of events on a trial is presented in Figure 1. A black central fixation point (0.36° × 0.36°) was presented at the start of each trial for 750–1200 ms (randomly selected at 50 ms increments). For half of the participants (n = 36), the fixation point was then replaced by a left- or right-pointing black double-headed arrow (2.98° × 1.48°). These participants were informed that the direction in which the arrow pointed was not predictive of the location at which the target would appear (*p* = 0.50). For the other half of the participants (n = 36) the cue was either a central black ‘M’ or ‘W’ symbol (1.49° × 2.41°), which were identical in shape and size. These participants were told that the identity of the letter informed them about which side of fixation that the target was likely to appear (*p* = 0.80). The symbol/target-side association was counterbalanced across participants. All cues remained present for the duration of the trial.

On each trial, the cue-target SOA was determined randomly from three equally likely SOAs of 100, 500, or 1000 ms. For each cue type, half of the participants (n = 18) completed the experiment under the ‘No Catch’ condition, in which a target appeared 100% of the time after the cue was presented. The target, a peripherally presented black ‘X’ (1.56° × 1.58°) onset randomly either 9.22° to the left or right of fixation. The participants’ task was to respond as fast and as accurately as possible by pressing the spacebar on the keyboard once the target was detected. The target remained on screen for 200 ms after a response, or for 3000 ms if there was no response. The remaining half of participants completed the experiment under the ‘Catch’ condition, whereby on 25% of the trials a target failed to appear after the cue. Participants were instructed to withhold a response on catch trials. On Catch trials, the trial ended 1250–1700 ms (randomly selected at 50 ms increments) after the onset of the cue. Trials were separated by an inter-trial interval of 500 ms.

In the arrow cue condition, each participant completed 10 practice trials, followed by three or four blocks of 100 trials (for No Catch, and Catch conditions, respectively) such that all participants received 300 target-present trials in which a response was required. In the letter cue condition, the overall trial number was increased to ensure that we collected a sufficient number of invalid trials, i.e., ~80. In the No Catch condition, a total of 420 test trials were collected, and a total of 520 test trials were collected in the Catch condition, meaning that 130 catch trials were included to maintain the same 25% no-target rate as was used in the arrow cue condition. These were divided into four or five blocks (No Catch and Catch, respectively). As before, each condition was preceded by 10 practice trials. Table 1 describes the probability of a target appearing following each SOA used. Trials in which the participant responded before the target onset, did not withhold a response in a no-target trial, or did not make a response within 3000 ms were recycled and presented again within the same block of trials.

## 3. Results

Overall, we found clear differences in RTs for Catch and No Catch trials, as well as distinct response patterns using spatially non-predictive arrows or spatially predictive letters as cues. In the absence of catch trials, the cue’s utility as a temporal signal of the upcoming target is maintained, and the foreperiod effect was observed for both arrows and letters. In contrast, when catch trials were interspersed into the blocks, the foreperiod effect was eliminated at later SOAs, i.e., RTs stabilized between SOAs of 500 ms and 1000 ms, demonstrating a lack of reliance on either the arrow or letter as a temporal predictor of the upcoming target.

Additionally, the manipulation of the cue’s temporal reliability also influenced spatial cuing magnitudes. At longer SOAs (500 and 1000 ms), there was spatial cuing observed in response to the spatially informative letter cue, regardless of whether catch trials were presented. However, when a spatially non-predictive arrow cue was presented, cuing effects were only observed when catch trials were absent in the study, i.e., when the cue was temporally informative.^1^

**Figure 2:**
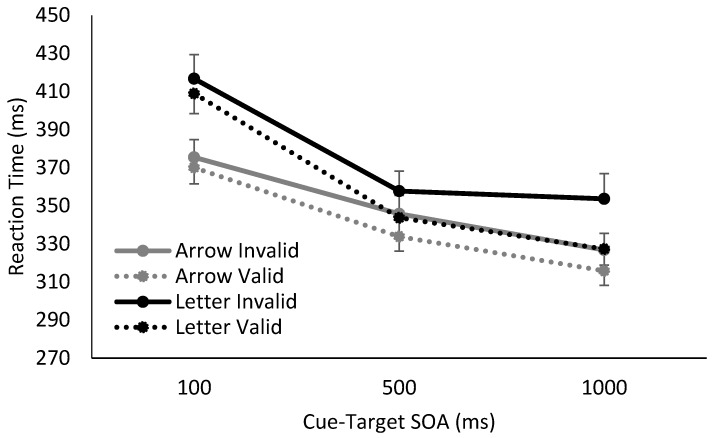
Reaction time results from when catch trials were absent, using either arrow or letter cues. Responses to both arrow and letter cues showed a foreperiod effect due to the temporal utility of the cue. Additionally, arrow and letter cues also produced small but significant cuing effects at SOAs of 500 and 1000 ms.

**Figure 3:**
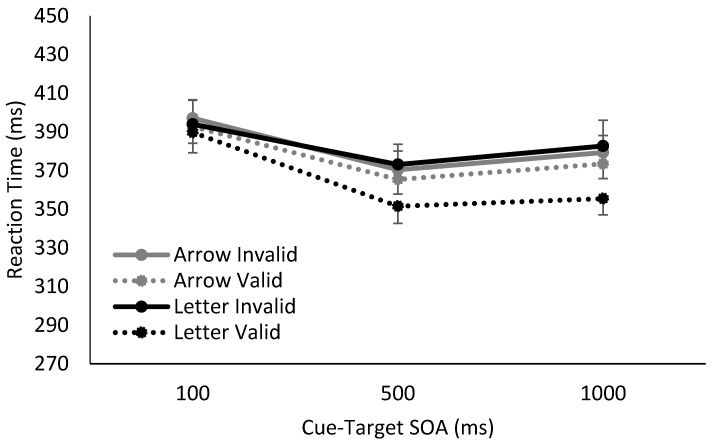
Reaction time results from when 25% trials were catch trials (i.e., contained no-target), using either arrow or letter cues. The introduction of catch trials eliminated the foreperiod effect on RTs for both arrow and letter cue conditions at later SOAs. When a letter cue was used, significant volitional spatial cuing was observed. In contrast, when the temporal utility of the arrow cue was disrupted by the inclusion of 25% catch trials, no significant reflexive spatial cuing effect was observed.

The results and follow-up analyses from mixed-effects analyses of variance (ANOVA) support these observations, and can be seen in Figure 2 and Figure 3. We divided the data based on whether catch trials were present or absent, and ran two separate mixed-factor ANOVAs with cue validity (invalid, valid) and SOA (100, 500, 1000 ms) as within-subject factors, and cue type (arrow, letter) as a between-subject factor. Relevant degrees of freedom were adjusted if Mauchly's test of sphericity was significant (conservatively set at α = 0.25; via Greenhouse–Geisser adjustment if ε ≤ 0.70, else via Huynh–Feldt adjustment). Follow-up multiple comparisons were Bonferroni corrected where appropriate.

In the absence of catch trials, there was a main effect of cue validity, a main effect of SOA, and a trending effect of cue type. There was a significant interaction between cue validity and SOA, (*F*(2,68) = 4.75, *p* = 0.01, η^2^_p_ = 0.12), such that the cues elicited significant spatial cuing at SOAs of 500 ms (*t*(35) = 3.05, *p* = 0.01, Hedges g_av_ = 0.33) and 1000 ms (*t*(35) = 3.66, *p* = 0.003, Hedges g_av_ = 0.45), but not at 100 ms (*t*(35) = 1.55, *p* = 0.39, Hedges g_av_ = 0.11). There was also a significant interaction between SOA and cue type, (*F*(2,68) = 10.73, *p* < 0.001, η^2^_p_ = 0.24), though there was nevertheless clear evidence of a sustained foreperiod effect for both letters and arrows, such that RTs continued to decrease between 500 and 1000 ms SOAs, (Arrow: *t*(17) = 4.52, *p* < 0.001, Hedges g_av_ = 0.52; Letter: *t*(17) = 4.26, *p* = 0.001, Hedges g_av_ = 0.35). Neither the interaction between cue validity and cue type, or the three way interaction between cue validity, SOA and cue type reached significance, all, *F* < 2.00, *p* > 0.15. To summarize, when a target was always presented, there was evidence of significant spatial cuing at 500 ms and 1000 ms SOAs for both letter and arrow cues, as well as a clear continued decrease in RTs as SOAs increased. There was no interaction between temporal attention and reflexive or volitional spatial attention.

Moving on to when 25% catch trials were included, interpretation of any significant lower-order main effects and interactions was qualified by the presence of a significant three-way interaction between cue validity, SOA, and cue type (*F*(2,68) = 5.93, *p* = 0.004, η^2^_p_ = 0.15).

To explore this three-way interaction, follow-up two-way mixed factor ANOVAs were conducted at each SOA with cue validity and cue type as factors. When catch trials were present and the SOA was 100 ms, no main effects or interactions between cue validity and cue type reached significance, all *F* < 2.50, *p* > 0.05. However, once SOA was increased to 500 and 1000 ms, there was a significant effect of cue validity, (500 ms: *F*(1,34) = 10.07, *p* < 0.003, η^2^_p_ = 0.23; 1000 ms: *F*(1,34) = 15.44, *p* < 0.001, η^2^_p_ = 0.31), as well as an interaction between cue validity and cue type, (500 ms: *F*(1,34) = 3.91, *p* = 0.06, η^2^_p_ = 0.10; 1000 ms: *F*(1,34) = 6.43, *p* = 0.01, η^2^_p_ = 0.16). The main effect of cue type did not reach significance, (500 ms: *F*(1,34) = 0.20, *p* = 0.61, η^2^_p_ = 0.006; 1000 ms: *F*(1,34) = 0.40, *p* = 0.53, η^2^_p_ = 0.01).

For both 500 ms and 1000 ms SOAs, investigation of the interaction between cue validity and cue type revealed significant spatial cuing effects when letter cues were used (500 ms: *t*(17) = 2.84, *p* = 0.02, Hedges g_av_ = 0.51; 1000ms: *t*(17) = 3.55, *p* = 0.005, Hedges g_av_ = 0.68), but no significant spatial cuing effects generated from arrow cues (500 ms: *t*(17) = 1.42, *p* = 0.35, Hedges g_av_ = 0.13; 1000ms: *t*(17) = 1.70, *p* = 0.26, Hedges g_av_ = 0.17). In sum, when catch trials were introduced, thereby resulting in the implicit withdrawal of temporal attention (as evidenced by the elimination of a foreperiod effect), a significant spatial cuing effect was observed only for spatially-predictive letter cues, i.e., volitional attention. There was no spatial cuing effect for arrow cues, i.e., no reflexive spatial orienting.

Error rates were low (well below 5%) in all conditions, excluding any possible speed-accuracy tradeoff compromising the above analyses. Of the test blocks, trials were recycled if participants responded prior to target onset (1.84%, SD = 1.86%), after 3000 ms had passed in target present trials (0.01%, SD: 0.06%), or if no response was withheld during a catch trial (0.53%, SD = 0.42%). Trials were removed from analysis if RTs were less than 100 ms (0.18%, SD = 0.35%), greater than 1000 ms (0.29%, SD = 0.52%), or beyond 2.5 SD away from the participant’s mean RT (2.30%, SD = 0.80%).

## 4. Discussion

The present study examined how reflexive and volitionally oriented spatial attention is similarily influenced by implicit changes in the allocation of temporal attention. Spatially non-predictive arrow cues were used to reflexively direct spatial attention, whereas predictive letter cues were used to engage volitional shifts in spatial attention. We questioned how these spatial attention effects would be modulated by changes in the implicit orienting of temporal attention, based on whether a cue was reliably followed by a target (no catch trials present) or not (catch trials present).

In the absence of catch trials, results demonstrate that temporal attention was engaged, as evidenced by a robust foreperiod effect. Further, robust spatial cuing effects were observed for both reflexive and volitional shifts of attention at longer SOAs of 500 ms and 1000 ms. No spatial cuing effects were observed at the 100 ms SOA. With letter cues, this is unsurprising, as volitionally-generated spatial cuing effects are known to take time to develop [49,50,51]. Similarly, though several authors have reported significant spatial orienting effects to arrows at short SOAs [21,52], others have not [14,53].

In contrast, when catch trials were introduced on 25% of the trials, temporal attention was no longer entrained by the cue, as evidenced by the lack of a foreperiod effect at later SOAs. The question was whether this would result in similar or divergent effects on reflexive and volitional spatial attention. Replicating Hayward and Ristic [43], reflexive spatial attention was affected by a change in temporal attention, though in the present study the reflexive attentional cuing effect was abolished, whereas in their study the effect was accentuated. Of note, the authors had hypothesized that spatial and temporal attention would interact in a similar way to what was observed in the present study, such that when a spatially non-predictive cue also did not provide useful temporal information concerning the appearance of the target, both the cue’s temporal and spatial effects would be reduced. However, when it was unexpectedly found that spatial cuing effects were enhanced by including 25% no-target catch trials, they speculated that withdrawing attention from time may have served to release attentional resources to be committed to the spatial cue.

It is unclear exactly what differed between our own paradigm and that used by Hayward and Ristic [43] to elicit opposing effects, and we cannot rule out the possibility that the interaction between temporal and spatial attention is extremely sensitive to changes in context and individual strategies; our use of a different number of SOAs, and target probabilities may have impacted the directionality of the results. Ultimately, however, our data is most consistent with Hayward and Ristic’s original proposal that reducing the reliability of the temporal association between the cue and target would detrimentally affect spatial orienting to the arrow cue, as its utility as a spatial cue was based precisely on reliable task contingencies between the appearance of the cue and the target in space and time.

Regardless of this discrepancy, the data converge on the same conclusion—that reflexive spatial orienting to a directional central cue is modulated by strategic changes in temporal attention. Critically, however, this finding was not observed for volitional spatial attention. In other words, in answer to our research question—‘Will implicit changes in temporal attention have the same effect on reflexive and volitional spatial orienting?’—the answer is an unambiguous no.

When the utility of the temporal information of a central cue is implicitly reduced by frequently failing to predict the later appearance of a target, participants ignore the central cues temporal information and along with it, its spatial directionality. The result is that there is no spatial orienting to arrows when there is no clear association between its directionality and the subsequent target’s location. In contrast, when the central cue reliably predicts the location of a target (i.e., the letter cues) then even when the temporal information is reduced, participants continue to utilize its spatial information to volitionally orient attention to the cued/likely target location. Participants were informed that the letter cue was spatially predictive, and thus may have been motivated to attend to its spatial information, unlike with the non-predictive arrow cue.

It might be noted that dissimilar cuing effects occurred in Catch and No Catch trials across SOAs that shared similar target onset probabilities. For instance, both the 100 ms SOA No Catch condition and the 500 ms SOA Catch condition shared the same probability (*p* = 0.33) of a target appearing at that SOA. Despite this, cuing effects were not observed for the 100 ms SOA (for arrows and letter cues), but cuing effects were present for letter cues in the 500 ms SOA Catch condition. As noted above, at the 100 ms SOA, it is unlikely that there was sufficient time for cuing effects to emerge, which was certainly not the case at the 500 ms SOA. In short, given the well-known finding that spatial cuing effects develop and change over time [48,49], we generally caution against drawing conclusions that ignore this temporal factor [50].

Our finding that reflexive spatial orienting to arrow cues is modulated by implicit changes in temporal attention suggests that there is an important volitional component to this effect, a conclusion which is convergent with Hayward and Ristic [43,46], as well as recent work by O’Malley and Besner [22]. In this latter investigation, the researchers concluded that central arrow or gaze cues that implicitly convey directionality still need volitional attention to be directed towards them in order to trigger a spatial cuing effect. In their study, participants were presented with both an arrow and a gaze cue, and told to respond to the direction of either the arrow or the eyes depending on which auditory tone they heard prior to the start of the trial. The researchers found no evidence of automatic processing of the arrow cue when told to respond to the gaze cue, or vice versa, suggesting that although these two cues may non-volitionally orient spatial attention when presented on their own, this appears to be contingent on the participant first attending to the cue voluntarily.

The present results also complement research investigating inhibition of return (IOR). For example, Tipper and Kingstone [42] investigated whether IOR was sensitive to changes in the reliability of the cue to predict whether and when a target would appear, and similarly reported a reduction in the magnitude of IOR when the cue was made temporally unreliable through the inclusion of catch trials. Gabay, Chica, Charras, Funes, and Henik [54] have suggested that attentional IOR effects are influenced by the degree to which a cue is processed. In their study, when a cue was rendered uninformative of another target-specific task, IOR effects were reduced. Likewise, in the present study spatial and temporal cuing effects were eliminated when spatially non-predictive arrow cues were rendered temporally unreliable, which we suggest was due to participants not volitionally attending to the spatial component of the cue when its temporal utility was compromised.

The present data suggest that reflexive orienting to a directional cue, or what Ristic and Kingstone [26] call ‘automated symbolic orienting’ is contingent on some degree of volitional attention being implicitly directed to the cue. This was also suggested by Hayward and Ristic [43] as a possible explanation for why spatial cuing effects were enhanced when temporal attention was not implicitly entrained to the stimuli. A difference rests though in our proposal that when a cue no longer provides reliable temporal information about the target’s appearance, participants can choose to withdraw attention away from the spatial component of the cue. In contrast, Ristic and Hayward [43] suggest that the volitional attentional resources that were previously entrained to the cue’s temporal information are redirected automatically to process the cue’s spatial information. It is unclear what mechanism might serve this automatic redirecting of attentional resources from time to space. It is our speculation that the unexpected results reported by Ristic and Hayward may reflect a volitional strategy employed by participants. Whereas participants volitionally reduced processing the cue when its overall utility was reduced in both our own and other studies [42,54], there may have been a contextually unique feature of either the paradigm, protocol, or participants used by Hayward and Ristic [43] that elicited a different strategic readjustment of attentional resources from temporal to spatial aspects of the cue.

This interpretation fits nicely as well with that of Olk and colleagues [55] who concluded that cues like arrows, which possess some inherent or overlearned directionality, are especially sensitive to the commitment of volitional attention [resulting in superadditivity; see also [52,56]. What is novel to the present study is that arrow cues seem also to be sensitive to the withdrawal of strategic attention, such that choosing not to use an arrow cue’s temporal information also negates the use of its spatial information. Whether this sensitivity is especially acute for cues that support automated orienting is an intriguing issue for future investigations. While it is not clear what neural processes are involved in interactions between spatial and temporal attention to arrow cues, its permeability to strategic effects suggests that cortical, rather than subcortical, regions likely play a critical role. Ristic and Kingstone [26] point to the ventrolateral frontoparietal attention network as having a potential role in automated attentional orienting, especially to behaviorally relevant cues, such as arrows, as it includes areas involved in both responding to environmental contingencies as well as switching between attentional networks [57].

We hasten to emphasize that we are not proposing that automated symbolic orienting and volitional spatial orienting to a non-directional predictive cue (e.g., letter cues in the present study) engage the same mechanisms. There is a wealth of evidence to the contrary [49]. In the present study, we found that withdrawing attention from the temporal domain did not negate the ‘pure’ volitional spatial attention effect that is produced in response to the letter cues, a novel discovery that we had set out to investigate. Moreover, this resistance of spatial attention to an implicit change in temporal attention, and automated symbolic orienting’s sensitivity to it, converges with a series of recent studies by Ristic and colleagues [26,27], which argue that automated symbolic orienting is a qualitatively unique form of attention. For example, in Ristic and Kingstone’s [26] paradigm, participants completed a target detection task in which a spatially non-predictive arrow cue was paired with either a peripheral onset cue (to elicit reflexive, exogenous orienting) or a predictive centrally-presented number cue (to promote volitional, endogenous orienting). Both reflexive and volitional cues produced additive effects with the arrow cue, consistent with the hypothesis that arrow cues operate independently of either purely reflexive or volitional cues. The same conclusion was also supported using a more difficult target discrimination task [27].

## 5. Conclusions

We began our study by noting that recent work suggests that temporal attention, when it is explicitly cued, does not interact with volitional or reflexive spatial attention to a central cue. However, when temporal attention is implicitly manipulated by including a large percentage of catch trials and thereby discouraging participants from expecting a target at a particular time, then temporal attention and spatial attention—that is, reflexive spatial attention—interact. What was unknown however, was whether this interaction would also be observed when spatial orienting was volitional rather than reflexive. The present study addressed precisely this question and discovered that whereas reflexive spatial attention orienting is modulated by implicit changes in temporal attention, volitional spatial attention orienting is not. These data converge on the notion that reflexive shifts of attention are sensitive to strategic changes in attention [22] even when those attentional changes are in a non-spatial domain [43]. Moreover, they dovetail with the idea that reflexive spatial orienting to a central cue that has implicit directionality (e.g., arrows), and volitional spatial orienting that is engaged by a central predictive cue that does not possess any inherent directionality (e.g., a letter) will engage qualitatively distinct spatial attentional mechanisms [26,27]. In conclusion, the present study indicates that the ways in which attention to time and space combine must be of serious consideration going forward in visual attention research.

## Figures and Tables

**Table 1:** Probability of target appearing following various stimulus-onset asynchronies in both the Catch and No Catch conditions for both arrow and letter cue tasks.

SOA (ms)	Probability of Target Appearance	
	No Catch: No-Target Trials Absent	Catch: No-Target Trials Present
100	0.33	0.25
500	0.50	0.33
1000	1.00	0.50
Target absent	n/a	0.00

## Footnotes

1 The manipulation of the cue’s temporal reliability also appears to have impacted RT at the shortest SOA, with the longest RTs observed when the amount of cue information to be processed was greatest, i.e., when spatially predictive letter cues were also temporally informative.
